# Appendiceal collision tumors: case reports, management and literature review

**DOI:** 10.3389/fsurg.2023.1184322

**Published:** 2023-06-07

**Authors:** Giovanni Viel, Francesco A. Ciarleglio, Marco Frisini, Stefano Marcucci, Stefano Valcanover, Emma Bragantini, Mattia Barbareschi, Liliana Mereu, Saverio Tateo, Elettra Merola, Franco Armelao, Giovanni De Pretis, Marco Brolese, Nicola L. Decarli, Alberto Brolese

**Affiliations:** ^1^ Department of Surgery, Hepato-Biliary Surgery Unit, Santa Chiara Hospital, Trento, Italy; ^2^ Pathology Unit, Department of Clinical Services, Santa Chiara Hospital, Trento, Italy; ^3^ Department of Obstetrics and Gynecology, Santa Chiara Hospital, Trento, Italy; ^4^ Department of Gastroenterology, Santa Chiara Hospital, Trento, Italy; ^5^ Hepatobiliary and Liver Transplant Unit, University of Padua School of Medicine, Padua, Italy

**Keywords:** appendiceal tumors, collision tumor, low-grade appendiceal mucinous neoplasm, neuroendocrine neoplasm NEN, appendectomy

## Abstract

Appendiceal tumors are incidentally detected in 0.5% cases of appendectomy for acute appendicitis and occur in approximately 1% of all appendectomies. Here, we report two cases of appendiceal collision tumors in two asymptomatic women. In both cases, imaging revealed right-lower-quadrant abdominal masses, which were laparoscopically resected. In both cases, histological examinations revealed an appendiceal collision tumor comprising a low-grade appendiceal mucinous neoplasm and well-differentiated neuroendocrine neoplasm (NEN). For complete oncological control, right hemicolectomy was performed in one patient for the aggressive behavior of NEN; however, histology revealed no metastasis. The other patient only underwent appendectomy. No further treatment was recommended. According to the latest guidelines, exact pathology needs to be defined. Proper management indicated by a multidisciplinary team is fundamental.

## Introduction

Primary appendiceal tumors are rare entities in heterogeneous group of tumors, with an incidence of approximately 1.2 case per 100,000 people annually in the United States (1). They are most commonly found incidentally in a surgical specimen after appendectomy for acute appendicitis. However, their pathology and classification remain controversial. Hence, a new classification of these neoplasms was published in the World Health Organization (WHO) Classification of tumors, 5th edition, 2019 (2). Mucinous neoplasm and neuroendocrine neoplasm (NEN) are the most frequent benignant and malignant lesions (3).

When tumor components are composed by two adjacent, different but separate neoplasms from 2 different cellular lines, they are called collision tumor (3, 4). Appendiceal collision tumors are rare entities. Only 13 cases have been reported in the international literature to date. Here, we present two new cases comprising a low-grade appendix mucinous neoplasm (LAMN) and a well-differentiated NEN, which were managed differently.

### Case presentation 1

**Figure 1:**
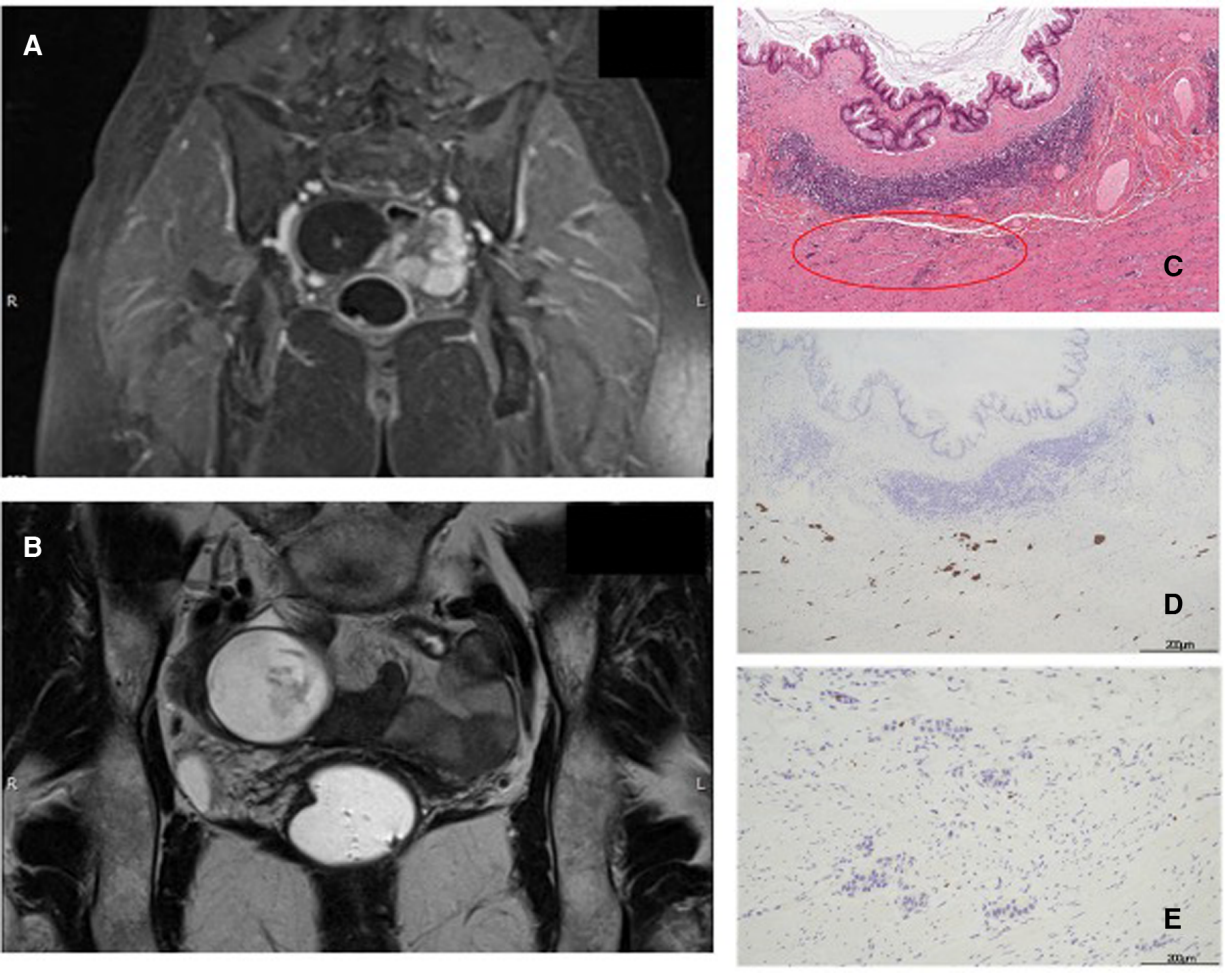
(**A,B**): Abdominal MR T1 and T2-weighted images. (**C**): LAMN and NEN (red circle), hematoxylin and eosin (H & E) staining 10×. (**D**): Immunohistochemistry positive for Cg A. (**E**): Immunohistochemistry for Ki-67: proliferation index of 0.4%.

A 49-year-old Caucasian woman with no significant medical history visited an ambulatory gynecology clinic for a routine check-up. Transvaginal ultrasonography revealed an oval mass with a mixed content measuring 74 mm × 44 mm in diameter, suggesting a dermoid cyst or an ovarian fibroma. Abdominal magnetic resonance imaging (MRI) also described a tumor close to the right ovarian gland (69 mm × 40 mm × 46 mm), with contrast enhancement in the arterial phase and clear margins afterward and a small nodulation inside (Figures 1A,B). Metastases or peritoneal deposits were not noted. Remarkably, Ca-125 and Ca 19-9 values were 10.7 and 42 U/ml (normal values: <35 and <37 U/ml), respectively. Thus, gynecologists performed laparoscopic surgery and found an appendiceal neoplasm intraoperatively. The surgery was completed with an appendectomy and a peritoneal biopsy performed by a general surgeon consultant. The specimen was removed through the umbilical port in an extraction bag, with no cystic lesion rupture. Intraoperative frozen sections indicated a LAMN. Macroscopically, the resected specimen showed an 8.5 cm-long appendix with a cystic neoformation measuring 8 cm × 5.5 cm × 5 cm, with mucinous content. At 1 cm proximal to the appendiceal cecal margin, another yellow node measuring 2.1 cm in diameter was detected. On histological examination, the bigger mass was described as a LAMN with acellular mucus confined to the wall (TNM Classification 8th edition 2016: pTis), whereas the smaller nodule was described as a NEN G1, characterized by mesenteric fat and visceral serous membrane involvement measuring 0.9 and 0.5 mm, respectively (Figure 1C). Perineural tumoral invasion without angiolymphatic invasion was observed. Immunohistochemical analysis revealed positivity for cromogranin A (Cg A) (Figure 1D) and synaptophysin, with a Ki-67 proliferation index of 0.4% (Figure 1E) (TNM Classification 8th edition 2016: pT2G1). Moreover, peritoneal biopsy was negative for tumor seeding. No complications occurred, and the patient was discharged on postoperative day (POD) 4. A multidisciplinary team analyzed the case and decided to perform segmental colectomy with lymph node dissection Finally, robot-assisted right hemicolectomy was performed. On POD 5, the patient was discharged after a regular postoperative course. Histologically, the specimen had no residual tumor and no nodal involvement (19 nodes). No adjuvant therapy was recommended. At 6 and 12 months follow-up, total body CT scan and assessment of serological markers showed no evidence of recurrence.

### Case presentation 2

**Figure 2:**
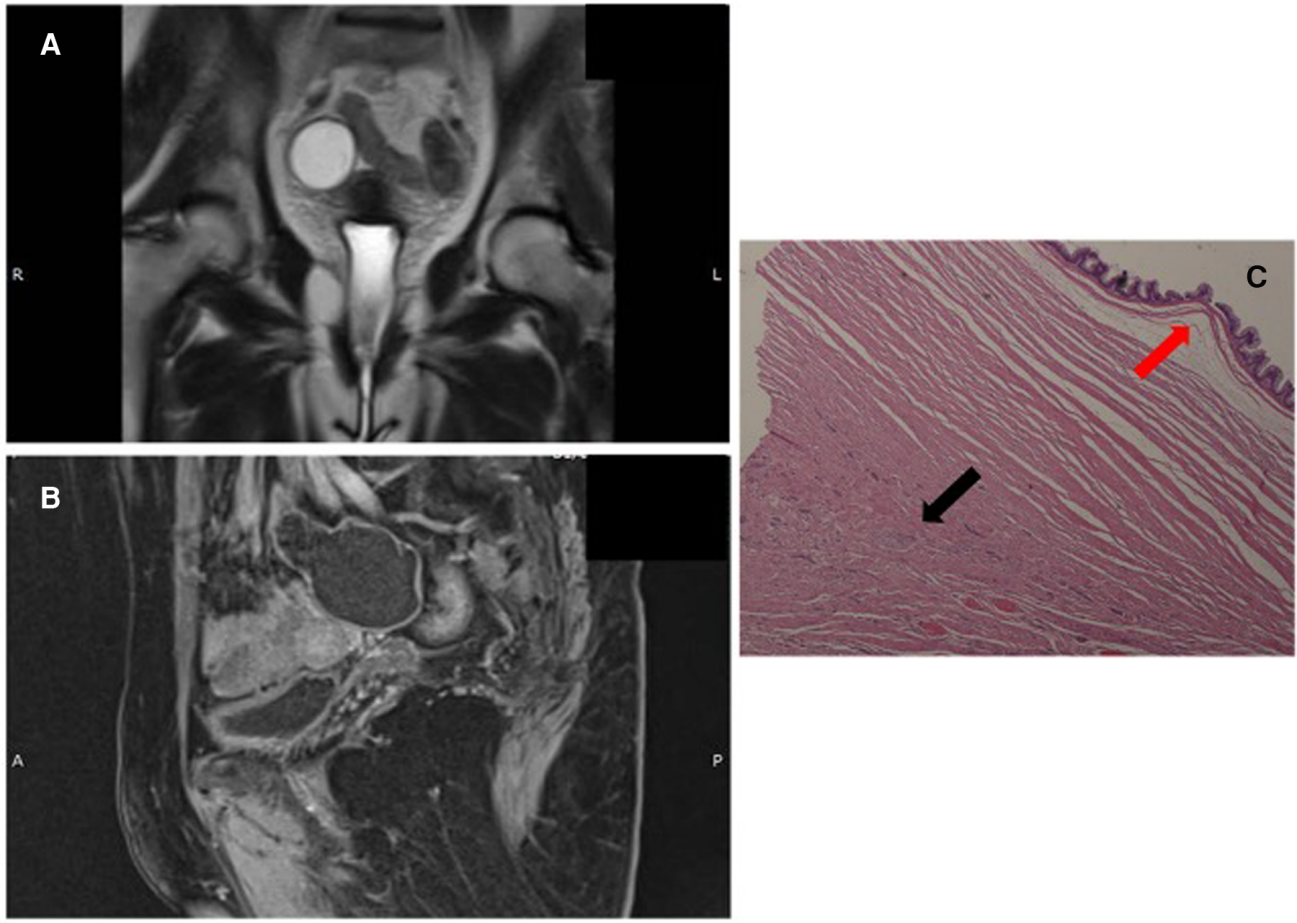
(**A,B**): abdominal MR T2- and T1-weighted images. (**C**): LAMN (red arrow) and NEN G1 (black arrow), (hematoxylin and eosin (H & E) staining).

A 59-year-old Caucasian asymptomatic woman underwent an abdominal ultrasound which revealed a right pelvic mass. Her Ca-125 value was 3.2 U/ml (normal value: < 35 U/ml). Abdominal MRI revealed a cystic oval mass [diameters 3.7 cm × 4.5 cm × 6.4 cm; hyperintense in T2-weighted images (Figure 2A) and hypointense in T1-weighted images Figure 2B)] in the right uterus space. Final radiological diagnosis was hydrosalpinx. Hence, the patient underwent laparoscopic surgical treatment. A general consultant surgeon performed appendectomy and appendiceal tumor (diameter 5 cm) with a smooth surface and stretched elastic consistency was found. No pelvic organ was involved. Subsequently, the patient demonstrated no complications, and on POD 2, she was discharged.

Gross morphology of the resected specimen showed a 9 cm-long cyst-like dilated appendix measuring 6 cm in diameter. The appendix was filled with thick mucus. Histologically (Figure 2C), the specimen appeared to be a LAMN with a fully thick mucus on the appendicular wall, but no peri-appendicular adipose tissue was involved (TNM Classification 8th edition 2016: pTis). A NEN G1 (9 mm × 7 mm) limited to the muscularis layer was identified in the proximal section of the appendix, with no serous and perivisceral fat invasion and no vascular or perineural neoplastic invasion. However, on immunohistochemical evaluation, Cg A and synaptophysin were positive. The Ki-67 proliferation index was 1% (TNM Classification 8th edition 2016: pT1G1). Additionally, the specimen had negative surgical margins. The multidisciplinary team did not recommend any adjuvant therapy. At 6 and 12 months follow-up, total body CT scan, abdominal ultrasound, and serological markers' assessment showed no evidence of recurrence.

## Discussion

Appendiceal tumors are extremely rare entities, usually detected incidentally following an emergent appendectomy for acute appendicitis in approximately 1% (1) of cases and occurring in approximately 1%–2% of all appendectomies (5).

Incidental diagnosis of asymptomatic patients in the course of another examination is relatively common, as noted in the two cases described.

According to the 5th edition of the WHO classification (2), appendiceal tumors are classified into several histological types, such as serrated lesions and polyps, mucinous neoplasms, adenocarcinomas, goblet cell adenocarcinoma, and NEN.

The mucinous tumors of the appendix are categorized into serrated polyps, hyperplastic polyps, LAMNs, high-grade appendiceal mucinous neoplasms (HAMNs), and mucinous adenocarcinomas (2, 6). Mucinous neoplasms are characterized by a dilated appendix containing luminal mucin. High secretion by these tumors can cause appendiceal rupture and tumoral cell dissemination in the peritoneal cavity. LAMNs are among the most common borderline neoplasms of the appendix, with an incidence of 0.3% in a recent series of appendectomy specimens (5). Histological examination show high-grade atypical glands with an infiltrative pattern extended through the muscularis mucosae. LAMNs comprise well-differentiated glands inside the muscularis mucosae, with dissecting mucin or epithelium and they do not exhibit infiltrative epithelial invasion of the appendiceal wall (2, 7–10).

Moreover, among the most common types of primary malignant lesion of the appendix are appendiceal NENs, with an incidence of approximately 0.15 per 100,000 people annually (11). The Ki-67 index determines the tumor grading according to the WHO and European Neuroendocrine Tumor Society classifications (2, 12). Generally, neuroendocrine tumors (NET) of the appendix are either G1 (more than 80%) (13) or G2 (14). These neoplasms appear as yellowish, well-demarcated nodules arising in any part of the appendix. Microscopically, they have uniform polygonal tumor cells frequently arranged in large nests (2).

Collision tumors results from the proliferation cellular lines. They are two distinct but adiacent neoplasms, retaining a transition between the two. Otherwise, a multidirectional differentiation of cells from a single tumor results in a combined neoplasm (3, 4).

The association between mucinous and neuroendocrine appendiceal tumors is an uncommon event with only few cases described (15). We found only 13 cases in 10 papers on PubMed research (Tables 1A,B). Our cases are appendiceal collision tumors, because both showed histologically distinct type of neoplastic cells with epithelial and neuroendocrine origin occurring in the same region the components, although intimately juxtaposed, are not intermixed and do not show transition, consistent with Singh NG et al.'s definition (16). The first case was of a LAMN containing acellular mucus confined to the wall; it was associated with a smaller NEN G1 nodule with mesenteric fat and visceral serous membrane involvement measuring 0.9 and 0.5 mm, respectively (Figure 1C). Perineural tumoral invasion without angiolymphatic invasion was also evident. In the second case, histological examination (Figure 2C) showed a LAMN with fully thick mucus on the appendicular wall; however, we did not observe the involvement of periappendicular adipose tissue associated with NEN G1, which was limited to the muscularis layer without serous, perivisceral, and vascular invasion. The mean age at diagnosis of patients with appendiceal collision tumors is 43 ± 12 years (23–60 years), with prevalence in women (8/5).

**Table 1A:** Literature review.

Authors	M/F	Age	Signs and/or symptoms	US	CT	MRI	CEA	Others markers	Appendectomy	Minimally invasive surgery	Right hemicolectomy
Tan (15)	F	59	Yes	na	na	na	na	na	Yes	Yes	No
	M	52	No	No	Yes	No	H	na	Yes	Yes	No
Baena-del-Valle et al. (26)	F	49	No	No	Yes	No	na	na	Yes	No	na
	M	45	Yes	na	na	na	na	na	Yes	na	na
Dellaportas et al. (20)	F	57	Yes	Yes	Yes	No	na	Normal	Yes	Yes	Yes
Singh (16)	M	52	Yes	Yes	Yes	No	H	na	No	No	Yes
Rossi et al. (33)	F	35	Yes	Yes	No	No	na	na	Yes	No	Yes
Sholi (25)	F	23	Yes	No	Yes	No	na	na	Yes	Yes	Yes
Ekinci (34)	M	60	Yes	Yes	No	No	H	Normal	Yes	na	No (patient refused surgery)
Sugarbaker (32)	F	39	Yes	Yes	No	No	na	na	Yes	No	Yes
	M	32	Yes	No	Yes	No	na	na	Yes	No	Yes
Cafaro et al. (35)	F	35	Yes	Yes	No	No	No	No	Yes	No	No
Villa et al. (3)	F	31	Yes	Yes	Yes	Yes	na	na	Yes	Yes	Yes
Present serie	F	49	No	Yes	No	Yes	na	H	Yes	Yes	Yes
	F	59	No	Yes	No	Yes	No	Normal	Yes	Yes	No

**Table 1B:** Literature review.

Authors	Pathology 1	Pathology 2	Ki-67%	FU months	Adjuvant therapy	Recurrence	Death
Tan (15)	Mucinous adenoma	NEN	na	60	No	No	No
	LAMN	NEN	na	3	No	No	No
Baena-del-Valle et al. (26)	LAMN	NEN	na	na	No	No	na
	LAMN	NEN	na	na	Yes	Yes	na
Dellaportas et al. (20)	Mucinous cystadenoma	NEN	na	12	No	No	No
Singh (16)	Adenoca	NEN	na	14	Yes	Yes	Yes
Rossi et al. (33)	Adenoca	NEN	na	65	Yes	No	No
Sholi (25)	LAMN	NEN	8	24	No	No	No
Ekinci (34)	LAMN	NEN	<1	6	No	No	No
Sugarbaker (32)	LAMN	NEN	na	60	Yes	Yes	No
	LAMN	NEN	5	12	Yes	No	No
Cafaro et al. (35)	LAMN	NEN	5	15	No	No	No
Villa et al. (3)	LAMN	NEN	<1	12	No	No	No
Present serie	LAMN	NEN	0.4	12	No	No	No
	LAMN	NEN	1	12	No	No	No

M, male; F, female; US, ultrasonography; CT, CT scan; MRI, magnetic resonance imaging; Adenoca, adenocarcinoma; CEA, carcinoembryonic antigen; LAMN, low-grade appendiceal mucinous neoplasm; NEN, neuroendocrine neoplasm; other markers, tumor markers generically reported in the papers; H, high; FU, follow-up (months) after surgery; na, not available.

Clinical presentation is not specific and is characterized by a wide spectrum of findings and symptoms. Patients may have specific symptoms of clinical acute appendicitis or colorectal carcinoma syndrome or even nonspecific symptoms. The diagnosis is usually made incidentally in the course of another examination. Our patients did not report any symptoms, including NEN-related symptoms (weight loss, diarrhoea, or cutaneous flushing).

The role of tumor markers is still insufficiently defined. An elevated serum carcinoembryonic antigen (CEA) level was reported in 3 cases of the literature (15, 16, 34). In our study, only Case 1 had slightly elevated CA 19-9 levels.

Preoperative diagnosis of appendiceal collision tumor is often incidental because this entity has no special radiological or clinical features (17). An eventual preoperative biopsy generally detects only one histological component, and it may only identify a mixed histology in only one-third of cases (18). Incidental radiological findings of a pelvic mass could be the first evidence of the disease in asymptomatic patients. CT scan is the gold standard preoperative diagnostic imaging test; it shows a cystic mass of liquid density adjacent to the caecum and at a retrocecal location in most cases (19). Unfortunately, mass dimensions and radiological characteristics on CT scan and MRI in some cases cannot identify the origin of tumors, particularly if the origin is ovarian or appendicular (20). In both our cases, radiological findings were compatible with both origins, and the final evidence of an appendiceal disease was determined only during surgery.

Gold standard treatment is surgery for selected case. Laparoscopic approach appears to be a safe and feasible option for not advanced cases (15). Appendectomy alone is the treatment of choice when benign lesions, such as adenoma or LAMN with negative margins and NEN of <1 cm, are present (21–23). In adenocarcinoma or NEN of >2 cm with the involvement of the appendiceal base, segmental colectomy with lymph node dissection for tumor staging is indicated (5, 21, 25). Right hemicolectomy should also be considered in NEN of 1 cm–2 cm with serosal involvement, Ki-67 proliferative index of >2%, location at the base of the appendix, and angioinvasion or neuroinvasion (5, 12, 21–25).

Initially, we performed a laparoscopic appendectomy with peritoneum biopsy in one case. Through the laparoscopic exploration, a pseudomyxoma peritonei was excluded. Postoperative morbidity was not observed. The effect of two different histological components increases the complexity of therapeutic approach because it is not yet clear whether biological behavior depends on a larger or more aggressive component (17).

Histologic findings are relevant to the prognosis and treatment of patients, and the management of collision tumors is guided by component neoplasms (25). Generally, the more aggressive histological pattern determines the clinical evolution of the disease (26). Duffy et al. (27) suggested that the treatment should be more aggressive in a collision tumor with major neuroendocrine components and high grading. Therefore, in relation to the pathology, we performed a simple appendectomy in one case and a minimally invasive right hemicolectomy for the more aggressive behaviour of NEN in the other case.

The most important factor for improving the outcome is early and accurate diagnosis with adequate histopathological examination to confirm the presence of two components within the same neoplasm (28). Immunohistochemical tests are the cornerstone in identifying a large number of these tumors, from adenomas or adenocarcinomas with several neuroendocrine cells to classical neuroendocrine tumors with focal exocrine/epithelial elements (17).

Moreover, adjuvant chemotherapy for collision tumor has not been evaluated in prospective randomized trials. Adjuvant chemotherapy is not recommended for low-grade, well-differentiated mucinous tumors and should only be considered in cancers with invasive features such as lymphovascular or lymph node involvement (29). Prevention or delayed neuroendocrine syndrome is not supported by randomized evidence from the perioperative setting of pure G2 or G3 NENs (30). However, advanced appendiceal NEN treatment with somatostatin analogs (SSAs) as the first-line approach is associated with more prolonged progression-free survival; however, in patients with a progressive disease despite receiving treatment with SSAs, further therapeutic modalities may include temozolomide-based chemotherapy (30, 31).

In previous studies, recurrent disease was only found in 3 patients with metastasis at the first operation (16, 26, 32, 33).

Long-term surveillance and follow-up are necessary for both tumor types according to final pathological reports. However, there are no suggested guidelines for an optimal postoperative follow-up (15).

## Conclusions

Appendiceal collision tumors are rare diseases; therefore, they continue to be challenging for physicians. Unfortunately, the small sample size of this study does not allow for definitive conclusions to be made. Considering the controversy relating to its deﬁnition, the limited diagnostic ability of biopsies, and the lack of awareness of this diagnosis within the scientiﬁc community, the disease remains underestimated. Currently, no shared guidelines are available. Moreover, the definitive diagnosis can be achieved only after surgery because NEN could be overlooked during diagnosis because of its small dimension. Therefore, each patient must be managed case by case, and a multidisciplinary team, including gynecologists, surgeons, radiologist, oncologist and pathologists with expertise in NENs, is important for appropriate management of patients. This approach involves various health professionals from different organizations to provide utmost care and advanced treatment to patients based on latest available insights into the disease.

## Data Availability

The original contributions presented in the study are included in the article, further inquiries can be directed to the corresponding authors.
